# Reconstruction of Metagenome-Assembled Genomes from Aquaria

**DOI:** 10.1128/MRA.00557-21

**Published:** 2021-08-05

**Authors:** Cassandra L. Ettinger, Jordan Bryan, Sima Tokajian, Guillaume Jospin, David Coil, Jonathan A. Eisen

**Affiliations:** a Genome Center, University of California, Davis, California, USA; b Department of Evolution and Ecology, University of California, Davis, California, USA; c Department of Microbiology & Plant Pathology, University of California, Riverside, California, USA; d College of Agriculture and Life Sciences, Cornell University, Ithaca, New York, USA; e Department of Natural Sciences, Lebanese American University, Byblos, Lebanon; f AnimalBiome, Oakland, California, USA; g Department of Medical Microbiology and Immunology, University of California, Davis, California, USA; University of Southern California

## Abstract

Here, we report 11 metagenome-assembled genomes (MAGs) reconstructed from freshwater and saltwater aquaria, including representatives of *Polynucleobacter*, *Anaerolinea*, *Roseobacter*, *Flavobacteriia*, *Octadecabacter*, Mycobacterium, and Candidate Phyla Radiation (CPR) members. These MAGs can serve as a resource for aquatic research and elucidating the role of CPR taxa in the built environment.

## ANNOUNCEMENT

Microbial communities play critical roles in aquarium health. Aquaria support complex multitrophic interactions between fish, invertebrates, plants, and microbial communities that occur in an enclosed built environment. Understanding the genomics of aquarium microbial communities is critical for understanding the health of other enclosed aquatic systems.

Samples were collected prior to the start of an undergraduate research project that investigated microbial community assembly of multiple aquaria in the fall of 2012 at the University of California, Davis (1). Tropical tank sediment (*n* = 3), cold reef tank sediment (*n* = 1), freshwater tank sediment (*n* = 3), cold reef tank water (*n* = 3), freshwater wipes (*n* = 3), and freshwater tank water (*n* = 3) were collected and processed for DNA extraction as described by Bik et al. (1). Libraries were made using a Nextera XT DNA library sample preparation kit (Illumina, Inc.) and were sequenced on an Illumina MiSeq instrument (paired end, 150-bp reads).

Reads were not quality filtered prior to assembly. All raw reads from all samples were coassembled using MEGAHIT (2) v.1.0.6. Metagenome-assembled genomes (MAGs) were generated using anvi’o v.2.3.2 (3). First, a contig database was produced using “anvi-gen-contigs-database,” and open reading frames were identified with Prodigal (4) v.2.6.2. We then used “anvi-run-hmms” to run HMMER v.3.1b2 (5) to identify bacterial (6) and archeal (7) single-copy genes. Contig taxonomy was inferred using Kaiju v.1.5.0 (8) with the NCBI BLAST nonredundant protein database, including fungi and microbial eukaryotes v.2016-09-18. Reads were mapped using Bowtie 2 v.2.2.8 (9) and SAMtools v.1.4.1 (10). Using “anvi-profile” and “anvi-merge,” contigs of >2.5 kbp were mapped to samples and then profiles were combined. On average, 780,565 reads per sample mapped to the contig database with the majority of mapped reads from cold reef tank water (57.3%) and freshwater tank water (41.7%). Contigs were clustered using “anvi-cluster-with-concoct” to automatically bin MAGs (11). MAG completeness and contamination were assessed in anvi’o using “anvi-summarize” and confirmed with CheckM v.1.0.7 (12). PhyloSift v.1.0.1 (13) was used to place MAGs in a phylogenetic context to provide additional information about taxonomic assignments. Candidate Phyla Radiation (CPR) genomes were identified with “anvi-script-gen-cpr-classifier” and “anvi-script-predict-cpr-genomes” using the Brown et al. (14) and Campbell et al. (6) databases. CPR genome completion was then re-estimated using 43 single-copy marker genes, as CPR members are known to have missing single-copy genes (14). CPR is putatively a diverse group of uncultured bacterial lineages with poorly understood metabolic functions known mostly from metagenomic sequencing work. Representatives of CPR have been previously found in a wide range of aquatic habitats, including bioreactors, ocean, lakes, groundwater, and waterways (15–21).

Using the standards suggested by Bowers et al. (22), we report two high-quality draft MAG sequences with >90% completion and four medium-quality draft MAG sequences with >70% completion (Table 1). Additionally, we report five high-quality draft MAG sequences that were identified as potential CPR genomes with >90% completion (Table 1). These metagenome-assembled genomes will enable deeper insights into the ecology of aquarium microbial communities and also into the possible functional roles of understudied lineages (e.g., CPR members) in the built environment.

**TABLE 1:** Genomic feature summary for metagenome-assembled genomes identified from aquaria

Bin identifier	Draft sequence quality^*a*^	Putative taxonomy	Genome size (bp)	No. of contigs	*N*_50_ (bp)	GC content (%)	No. of genes^*b*^	CheckM % completion	CheckM % redundancy	anvi’o % completion	anvi’o % redundancy	CPR % completion^*c*^	GenBank accession no.
AQU-01	High	*Polynucleobacter* sp.	1,676,117	78	37,546	45.38	1,729	97.69	0.16	98.56	0.72	NA^*d*^	JAHBAK000000000
AQU-02	High	*Anaerolinea* sp.	5,354,340	574	12,300	53.21	4,854	91.36	1.73	95.68	2.88	NA	JAHBAL000000000
AQU-03	Medium	*Roseobacter* sp.	2,693,064	264	12,778	60.69	2,712	88.8	0.63	75.54	2.16	NA	JAHBAM000000000
AQU-04	Medium	*Flavobacteriia* sp.	1,846,950	228	9,847	41.36	1,796	86.62	0.07	92.81	1.44	NA	JAHBAN000000000
AQU-05	Medium	*Octadecabacter* sp.	2,609,161	434	6,891	55.83	2,896	84.33	1.96	76.98	1.44	NA	JAHBAO000000000
AQU-06	Medium	Mycobacterium sp.	3,351,002	625	5,981	66.59	3,663	70.94	1.74	71.94	2.16	NA	JAHBAP000000000
AQU-07	High	Candidatus *Shapirobacteria* sp.	863,951	104	11,221	35.61	941	77.27	1.72	76.98	3.6	93.02	JAHBAQ000000000
AQU-08	High	Candidatus *Kerfeldbacteria* sp.	1,070,839	48	32,721	46.2	1,043	72.94	0.31	84.89	2.88	93.02	JAHBAR000000000
AQU-09	High	Candidatus *Uhrbacteria* sp.	1,115,436	46	43,390	51.31	1,073	68.81	0.5	77.7	0	93.02	JAHBAS000000000
AQU-10	High	Candidatus *Moranbacteria* sp.	892,370	24	56,939	43.56	901	67.95	0.99	82.01	0	93.02	JAHBAT000000000
AQU-11	High	Candidatus *Saccharibacteria* sp.	1,090,569	48	32,149	47.69	1,151	56.94	1.03	71.22	0.72	90.7	JAHBAU000000000

a Quality estimates were based on CheckM values for non-CPR members and the CPR-specific completion estimates for CPR members.

b No. of genes predicted by Prodigal.

c Completion estimates were generated using 43 single-copy markers for CPR members following Brown et al. (14).

d NA, not applicable.

### Data availability.

The raw sequencing reads, the coassembly, and the individual MAGs were deposited at DDBJ/ENA/GenBank under BioProject accession number PRJNA728121. Contigs identified as possible contaminants or adaptors by the NCBI Contamination Screen were subsequently trimmed or removed from the coassembly or individual MAGs during deposition.
